# Benchmarking DFT Accuracy in Predicting O 1s Binding Energies on Metals

**DOI:** 10.1021/acs.jpcc.5c05986

**Published:** 2025-10-08

**Authors:** Elizabeth E. Happel, E. Charles H. Sykes, Matthew M. Montemore

**Affiliations:** † Department of Chemistry, 1810 Tufts University, Medford, Massachusetts 02155, United States; ‡ Department of Chemical and Biological Engineering, Tufts University, Medford, Massachusetts 02155, United States; § Department of Chemical and Biomolecular Engineering, 5783 Tulane University, New Orleans, Louisiana 70118, United States

## Abstract

X-ray photoelectron spectroscopy (XPS) is a powerful tool for probing the electronic structure and composition of materials, particularly metals and metal oxides of relevance to solar cells and catalysis. Density functional theory (DFT) is often used to support XPS peak assignments, but its reliability for predicting oxygen species is not well established. Here, we compile a large data set of experimental oxygen binding energies and evaluate corresponding DFT predictions. We find that as the binding energies of metal-bound atomic oxygen species increase, especially above ≈530 eV, there is a general decrease in the accuracy of DFT-predicted values. Thus, high-binding-energy atomic oxygen species, such as those proposed as active for selective Ag-catalyzed epoxidation, are less well represented. The chemical nature of the oxygen species also influences accuracy, with molecularly bound species more reliably captured across the entire range of energies. These findings illustrate the limitations of DFT for interpreting XPS spectra and provide a benchmark for improving computational methods.

## Graphical abstract

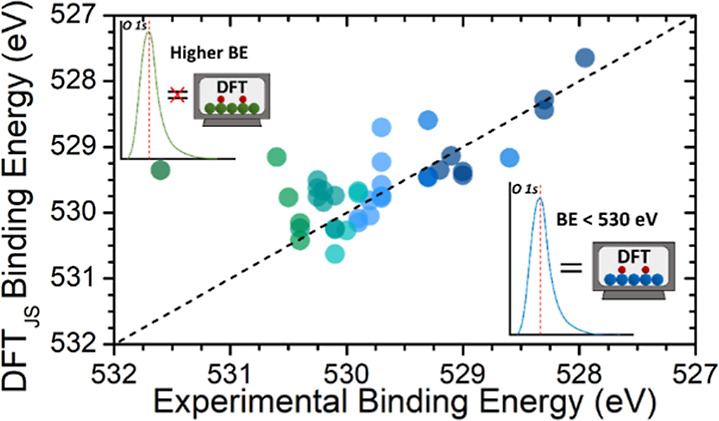

## Introduction

X-ray photoelectron spectroscopy (XPS) is a versatile and powerful analytical technique that provides critical insights into the elemental composition, electronic structure, and chemical state of atoms within a material. Due to its high utility and generally nondestructive nature, XPS has become one of the most widely utilized tools for investigating a broad range of materials, including semiconductors, organic compounds, thin film coatings, and catalytic surfaces.^1–7^ One distinguishing feature of XPS is its ability to operate under both ultrahigh vacuum (UHV) and near-ambient pressure (NAP) conditions, making it ideally suited for studying surface properties under different environments.

One of the primary challenges in interpreting XPS data lies in correlating the observed binding energies with specific atomic structures. Thus, while XPS yields critical insights into the elemental composition and electronic structure, interpretation often requires validation through complementary techniques.^8,9^ Methods such as low-energy electron diffraction (LEED), Auger electron spectroscopy (AES), and temperature-programmed desorption (TPD) contribute valuable information about surface order, composition, and reactivity. However, each of these techniques has its own limitations; for example, LEED requires ultrahigh vacuum conditions and highly ordered surfaces, restricting its use for amorphous and polycrystalline materials.^10^ Furthermore, dynamic surface changes, contamination, and nonequilibrated states further complicate the interpretation of XPS spectra.^11,12^

To address these challenges, density functional theory (DFT) has emerged as an indispensable tool for linking experimental XPS results with specific structural models. By predicting core-level binding energies (BEs) based on well-defined surface structures, DFT provides a computational framework to help deconvolute the ambiguities inherent in experimental measurements.^13^ However, DFT and other quantum mechanical predictions of BEs are an active area of research, and their accuracy is not always clear, particularly for oxygen.^14–20^ For some classes of materials, such as polymers and molecules, there have been relatively comprehensive studies on the accuracy of binding energy predictions,^21–23^ but the accuracy for O in metal-based systems is not clear, and some previous studies have found large errors for these systems.^19,20^

Transition metal and metal oxide systems are central not only to catalytic applications, such as selective partial oxidation reactions, but also to a range of other technologies, including solar cells and electronic devices. In these contexts, the synergy between experimental techniques such as XPS and computational approaches such as DFT enables a more comprehensive understanding of material behavior under operational conditions. One key example of this is the investigation of oxygen species on silver surfaces involved in partial oxidation reactions. While extensive studies have reliably characterized nucleophilic oxygen species forming well-ordered reconstructions on Ag(111), electrophilic oxygen species remain undefined, possibly due to their more disordered nature and/or challenges in computational modeling.^24^ Although silver-based systems serve as a compelling example, the insights gained from this study extend to a broader spectrum of metal/metal oxide materials.

In this work, we find a general pattern across many well-characterized surface systems: DFT performs well in predicting the binding energies of lower-energy nucleophilic oxygen species on metals, with strong agreement between experimental and theoretical values. However, as the binding energies increase, the agreement declines. The higher error in this regime, particularly above 530 eV, does not appear to be solely an artifact of binding energy, as DFT more reliably models higher binding energies of O in molecularly bound species such as CO, NO, and H_2_O on metal surfaces. Understanding and addressing these limitations are essential for improving the accuracy of DFT predictions and advancing the study of catalytic surface reactions.

## Methods

Density functional theory (DFT) calculations were performed using the plane-wave VASP code^25,26^ with the projector-augmented wave method^27^ for core electrons. The PBE functional was used with the Tkatchenko–Scheffler method for dispersion corrections.^28,29^ The energy cutoff was set to 400 eV. An energy convergence tolerance of 10^–5^ eV was used in all cases, and for relaxations, the geometric convergence tolerance was 0.03 eV/Å. For 3 × 3 surface cells, we used a 7 × 7 × 1 *k*-point grid, and for other cases, we calculated the number of *k*-points *n* in each nonvacuum direction as n=62Å/|a⃗| , where |a⃗| is the length of the corresponding lattice vector. We performed a few test calculations, which suggested that our calculated BE values do not change significantly (<0.04 eV) upon significantly increasing the *k*-point sampling density or energy cutoff, changing the O pseudopotential, or including spin–orbit coupling. For surface calculations, four layers were typically used, with the bottom two fixed at their bulk positions. To ensure that BE calculations were not overly influenced by small unit cells, supercells were created for BE calculations such that lattice vectors were typically larger than 8–10 Å. The relaxed structure for each case is provided separately in a VASP POSCAR format.

The Janak–Slater method was the primary focus of this work, which uses core-level eigenvalues after 0.5 electrons have been removed from the orbital. We also tested the JS­(0,1) method,^30^ which uses the equation $\epsilon \left(1\right)+\frac{1}{2}\left[\epsilon \left(0\right)-\epsilon \left(1\right)\right]$ , where ϵ(0) is the eigenvalue in the initial state and ϵ(1) is the eigenvalue in the final state (i.e., with 1 electron removed from the orbital). The final-state method uses the total energy difference between the initial state and the final state. Finally, we tested the use of the eigenvalues in the final state, which we denote as FS_Eigenvalue. All eigenvalues were referenced to the Fermi energy.

For Figures 1, 2, S1, and S2, we shifted the DFT predictions from their raw values as the raw values are not expected to be quantitatively accurate. Similar shifts have been performed in previous work,^24^ and we obtain similar BEs for the systems we have in common with this previous work. An alternative approach would be to calibrate based on a linear fit to all of the data.^31,32^ In our case, this would use systems with large errors as part of the calibration and decrease the accuracy for systems that seem to be well represented. Furthermore, this approach would require future studies testing advanced methodologies to perform a large number of calculations to directly compare with our work. Thus, we used the average of the O/Ag(111) and O/Pd(111) structures to shift the DFT predictions to the experimental values. This not only avoids relying on a single system but also focuses on the BE region where we find DFT to be more accurate while requiring only a small number of calculations to test other methodologies. For the Janak–Slater method, this corresponds to shifting all BEs down by 10.56 eV. Note that this correction has no effect on Figure 3, as that involves *R* ^2^ values of linear fits.

## Results and Discussion

To test the accuracy of DFT predictions of the O 1s binding energies, we first collected a data set from previous XPS studies of adsorbed oxygen, surface oxides, and bulk oxides, all for transition metals (Table 1). We include 14 host metal surfaces in both BCC and FCC crystal structures. Where possible, we included references with self-consistent structural and binding energy assignments from primarily experimental techniques. Some of these structures have near-identical reported values across publications, while others show some variation, mostly within a ±0.3 eV range, depending on the X-ray source; in these circumstances, the average BE of a given structure is given. A spreadsheet of these BE values is given in the Supporting Information, along with the DFT-relaxed structures.

**Table 1:** A Summary of XPS Results for Well-Investigated Adsorbed O, Surface Oxide, and Bulk Oxide Structures on Transition Metals^a^

host metal	facet/structure	bulk/surface	coverage (ML)	Avg BE
				
Au	Au_2_O_3_ ^33^	bulk		530.1, 529.0 ^34,35^
	(111) 2 × 2	surface	0.25	529.3 ^36–38^
				
Pt	(111) 2 × 2 ^39^	surface	0.25	529.9 ^39–42^
	PtO_2_ ^39^	bulk		530.2 ^39,43,44^
				
Ir	(111) 2 × 2 ^45^	surface	0.25	529.9 ^46–48^
	(110) 2 × 2 ^49^	surface	≈0.5	530.5 ^49–51^
	(100) 2 × 1 ^52^	surface	0.5	530.6 ^53^
				
Re	ReO_2_	bulk		530.1 ^54^
	(0001) 2 × 2 ^55^	surface	0.25	530.1 ^55–57^
	ReO_3_ ^58^	bulk		531.6 ^54,59^
				
Ag	(111) p(4 × 4)^60^	surface	0.375	528.3 ^60–63^
	(111) Ag_2_O^64^	bulk		529.0 ^65–67^
				
Pd	(111) 2 × 2 ^68^	surface	0.25	529.2 ^68,69^
	(110) c(2 × 4)^70,71^	surface	0.5	529.3 ^72,73^
	Pd_5_O_4_ ^74^	surface (overlayer)		529.5 ^69,74–76^
	PdO^77^	bulk		530.0 ^69,72,75,78^
				
Rh	(110) (2 × 1)p2 mg	surface	≈0.8	530.25 ^79^
	(100) p(2 × 2)^80^	surface	0.12–1	529.8 ^81^
	(100) c(2 × 8)^80^	surface		528.6, 529.7 ^82^
				
Ru	(0001) 2 × 2 ^83^	surface	0.25	529.8 ^84–86^
	RuO_2_ ^87^	bulk		529.3 ^88–90^
				
Cu	(110) 2 × 1 ^91^	surface	0.5	530.1 ^91–94^
	(100) 2√2×√2R45°^93^	surface	0.5	529.9 ^93,95,96^
	(111) 2 × 2	surface	0.25	529.7 ^97,98^
	(110) c(2 × 6)^91^	surface	0.666	529.7 ^91,94^
				
Ni	(100) c(2 × 2)^99^	surface	0.5	530.2 ^100–102^
				
Mn	Mn_2_O_3_	bulk		529.9 ^103–105^
				
Ti	TiO_2_ ^106,107^	bulk		530.4 ^106,108–110^
				

a See the text for a discussion of important considerations around the accuracy and reliability of BE values.

Some of these systems listed in Table 1 have been very well characterized. For example, the (2 × 2) O structure on Pt(111) has been extensively investigated, in no small part due to its catalytic relevance. However, for some structures, such as the p(4 × 4) O reconstruction on Ag(111), there is an agreement on the general structure and binding energy, but some studies have observed multiple coexisting structures on the same sample. We have also included surfaces with more conflicting reports of saturation coverage or other structural features, such as the (2 × 2) O structure on Cu(111). In these cases, we have assigned coverage arbitrarily, which is needed for DFT calculations. For further details on the data and how we chose to model the structures, see the Supporting Information.

There are inherent complexities that preclude the complete certainty of assignments of experimental BEs in many cases. For instance, even simple assignments in the literature can be inconsistent due to factors such as sampling depth. Although XPS is considered a surface-sensitive technique, it probes multiple atomic layers, which can introduce ambiguity when distinguishing between surface and bulk species. This issue is exemplified in ReO_3_, where conflicting reports suggest BEs of ≈529 and ≈531 eV, potentially reflecting contributions from both bulk and surface layers.^111,112^ Such discrepancies complicate direct comparisons and necessitate careful experimental validation.

Another critical consideration in BE measurements is the spectrometer calibration method. While workfunction shifts can be corrected using valence band spectra, it is common practice to reference BEs to the well-known “adventitious carbon”. This practice, while convenient, is not always considered best practice and has been cited as a potential risk for error in reporting XPS values because carbon can exist in several forms and many compounds.^113^ Consequently, minor and major differences in experimentally determined values are to be expected, and systematic computational benchmarking against well-characterized reference systems can be made difficult by inconsistencies in the experimental reports. Therefore, it is possible that some of the experimental values in Table 1 are simply inaccurate for one of these reasons. However, by taking into account the range of values in the literature that are associated with different calibration methods among other experimental inconsistencies discussed above, we expect that our data points accurately reflect accepted experimental values for these well-investigated oxygen/metal systems but should be understood in terms of the error associated with typical experimental results.

We next calculated the BEs for all of the structures using DFT. We used the PBE functional and the Janak–Slater method for calculating core-level BEs, which consisted of removing 0.5 electrons from the target orbital. This technique is not expected to give accurate absolute values for BEs; thus, we shifted the values based on O/Ag(111) and O/Pd(111) (see the Methods for more details). We focus on the Janak–Slater method in this work, in part, because of previous work suggesting it is accurate for gas-phase species, although we compare it to other methods below.^114^

We compare the experimentally determined O 1s BEs (Table 1) with DFT-calculated BE values (Figure 1A) and observe that lower BE species (<530 eV) exhibit strong agreement between experiment and theory, with most errors less than 0.3 eV in this region. However, at higher BEs (>530 eV), the deviation between DFT and experimental values increases significantly, with many errors over 0.5 eV and several over 1 eV (Figure 1B). While we chose 530 eV as the cutoff for this analysis, the deviation in fact appears to increase relatively smoothly as the binding energy increases. This trend suggests a systematic limitation in DFT predictions for oxygen species with higher experimental BEs. Thus, DFT is not generally a reliable predictor of experimental O 1s BEs (Figure 1C).

**Figure 1:**
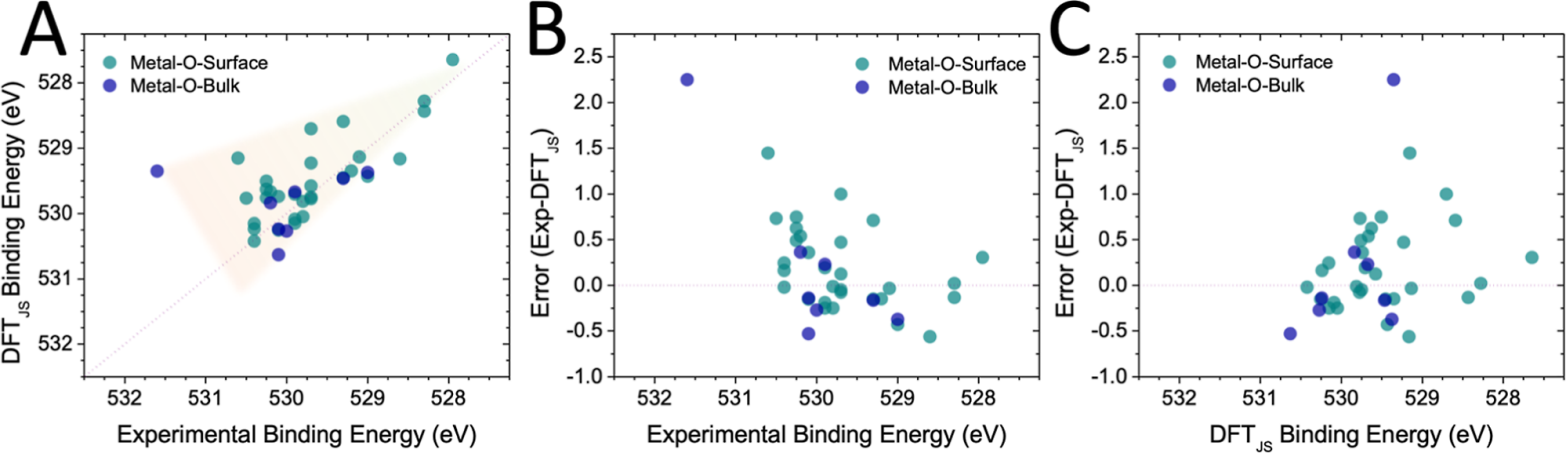
A comparison of experimental and DFT (Janak–Slater)-calculated BEs for atomic oxygen systems. Plotting experimentally determined BEs against DFT predictions (A) reveals a decrease in accuracy as the experimental binding energy increases. The difference/error associated with these calculations (B) shows that the error is notably increased in the higher binding energy range. The same error is plotted against the BEs determined by DFT (C), where the scatter is shifted due to DFT’s tendency to underpredict high BEs.

Interestingly, we find that DFT accurately predicts the BEs of oxygen-containing molecular adsorbates, such as CO, even in the high-BE range (Figure 2). We refer to these cases as “molecularly” bound, where the O is bound to C, H, or O. This observation indicates that the discrepancies are not merely a function of BE magnitude but rather reflect fundamental differences in the electronic structure.

**Figure 2:**
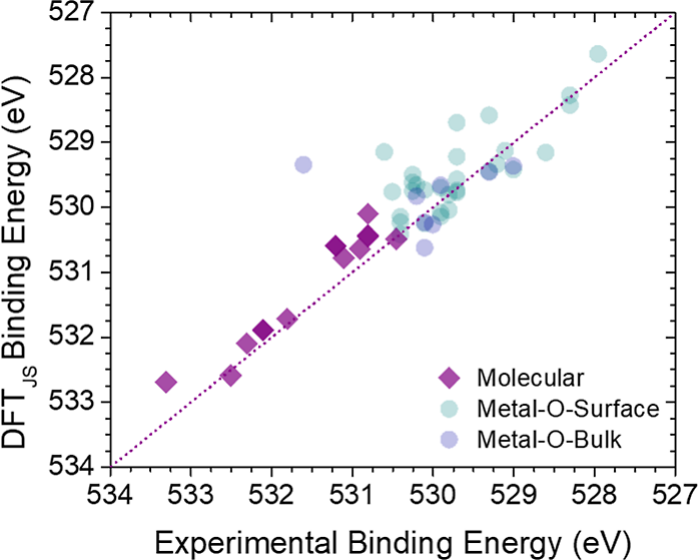
DFT predictions of BEs larger than 530 eV are more accurate for oxygen-containing molecular adsorbates on metal surfaces as compared to atomic O structures. DFT-calculated BEs vs experimentally determined BEs for oxygen-containing molecular species (purple diamonds) and various types of atomic oxygen species (circles).

Our data set also allows us to test the accuracy of multiple DFT BE modeling methodologies. We compared the initial-state approximation, the Janak–Slate approximation, and the final-state approximation (see Supporting Information for additional details). First, it is clear that DFT predictions for molecularly bound species show consistently lower errors than those for electrophilic oxygen species, as evidenced by the higher correlation (*R* ^2^) between experimental and theoretical values (Figure 3). This suggests that DFT’s inaccuracy for high-BE species is not easily fixed by a small change to the methodology. Second, we find that the Janak–Slater approximation, which uses core-level eigenvalues after 0.5 electrons have been removed from the orbital, and the final-state approximation, which uses the total energy difference between the initial and final states, gives quite similar accuracies. However, the initial-state approximation, which uses eigenvalues in the initial state, is less accurate. While previous studies have suggested that the initial-state approximation often gives accurate trends for metal atoms’ core levels, at least for a set of similar systems,^61,115–118^ it is clearly quite inaccurate for the broad set of O 1s core levels in our data set. We also tested two additional methodologies. The first, which we denote JS­(1,0), uses the O 1s eigenvalues in the initial and final states as an alternative approximation to the Janak–Slater method.^30^ The second, FS_Eigenvalue, uses the O 1s eigenvalues from the final state itself rather than the total energy difference between the initial state and final state. These are described in detail in the Methods section. Other than the initial-state approximation, all methods generally give similar *R* ^2^ values for each data subset.

**Figure 3:**
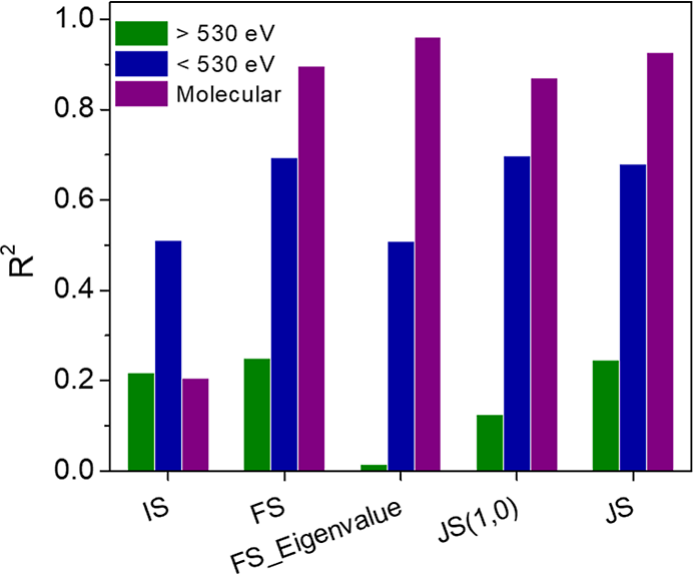
A comparison of methodologies for O 1s BE calculations. *R* ^2^ values for linear fits between DFT predictions and experimental values. Across several common DFT methods, the accuracy of DFT predictions for binding energies of O/metal surfaces remains similar. Apart from the initial-state approximation, molecularly bound oxygen species and lower binding energy structures are more accurately predicted by all methods.

Our results raise the question of why DFT is reasonably accurate for metal-bound atomic oxygen species with BEs generally less than 530 eV and for O bound in a molecule but not for metal-bound atomic oxygen species with increasing BEs. There are several common sources of error that are considered when addressing DFT predictions, with self-interaction error (SIE) being especially of note for core-level BEs. Empirically, the SIE can be larger for O in some states than others, with O_2_ and many metal oxides being cases where the SIE can lead to inaccurate total energies.^119–121^ We hypothesize that SIE may also affect predicted O 1s binding energies differently for different types of systems, leading to a regime in which predictions are poor. This could lead to the underestimation of experimental BEs seen in Figure 1, where even as experimental results yield a binding energy >530 eV for a given species, DFT consistently predicts a lower BE. This error might have a smaller effect on reconstructed systems that tend to have lower BEs, where charge may be distributed across the surface lattice, which may help mitigate some of the effects of SIE. However, there is precedent for SIE causing too much delocalization and underestimating BEs of covalent species, which we do not see in our investigation.^122–124^

Therefore, development of improved functionals for predicting reaction energetics—which are based on ground-state total energies—may not necessarily improve predictions for O 1s binding energies >530 eV. This is because the core electrons are in some ways qualitatively different from the valence electrons that are the prime drivers of chemical bonding. This is in addition to the intricacy of oxygen adsorption systems, such as coverage effects and other experimental complexities.

## Conclusions

Overall, our findings highlight the strengths and limitations of DFT in modeling core-level BEs for oxygen–metal systems. While DFT performs reasonably well for low-BE (<roughly 530 eV) oxygen and oxygen in molecular species, its accuracy decreases for high-BE (>roughly 530 eV) oxygen bound solely to metal atoms, likely due to fundamental limitations in current, commonly used exchange–correlation functionals. This could explain why previous computational studies that search for electrophilic oxygen on Ag have been unable to identify any strong candidates. This work also serves as a fundamental benchmark for future DFT investigations of the binding energies of oxygen species on metal surfaces for many research applications. Beyond this, alternative functionals or improved high-level methods should be investigated to improve the gap between experimental and computational values and optimized for improved predictive accuracy.

## Supplementary Material







## Notes

Additional description of methods (PDF)
Spreadsheet of experimental binding energies (XLSX)
Coordinates of structures in a VASP POSCAR format (ZIP)
