# Supplementary material for: Benchmarking DFT Accuracy in Predicting O 1s Binding Energies on Metals
Source: J Phys Chem C Nanomater Interfaces. 2025 Oct 8;129(42):19199–206. doi: 10.1021/acs.jpcc.5c05986 (PMC12557369; doi:10.1021/acs.jpcc.5c05986)
Supplement: Supplementary file 1 [file jp5c05986_si_001.pdf]

Supporting Information

Benchmarking DFT Accuracy for O 1s Binding Energies on Metals

Elizabeth E. Happel[1], E. Charles. H Sykes[1,2], and Matthew M. Montemore[3*]

[1]Department of Chemistry, Tufts University, Medford, MA 02155, United States.

[2]Department of Chemical and Biological Engineering, Tufts University, Medford, MA 02155, United States.

[3]Department of Chemical and Biomolecular Engineering, Tulane University, New Orleans, Louisiana 70118, United States

***Corresponding Author:** mmontemore@tulane.edu

Data Collection and Processing

Where possible, references where surface structure and binding energy were determined concurrently were prioritized. For surfaces where XPS measurements were consistently taken independently of specific structural measurements without any instances of self-contained structural and binding energy data, supplemental structure-only references are included and are then solely referenced in the *Structure/Facet* columns of each table. Most species have been validated structurally with explicit structural references; however, some were described only through standard naming practices (e.g. (2×2) structure on Au(111)). Priority was given to samples with explicit characterization in addition to BE measurements and we therefore report that in many cases experimental results are not based on pristine samples of only one species and as such there is an expectation for some variety in resolution and FWHM.

For some cases, we chose a coverage for the DFT model even though the coverage was somewhat unclear or varied in the experimental work; specifically: Ir(110) (2×2), Rh(100) c(2×8), and Rh(110) (2×1)p2mg. These cases typically did not give large errors, suggesting our choices were reasonable.

The structures for two bulk materials were collected solely from the Materials Project rather than direct experimental references: ReO_2 and Mn_2O_3.[1] We relaxed these structures prior to calculating the BEs. Both materials have been well characterized by XPS and have good agreement between DFT-calculated and experimental binding energies.

Finally, we have taken into account the change in assignments of specific binding energy features in literature over time, specifically with the Ir(100) (2×1) surface. There are some disagreements about the assignment of two features in the O 1s spectra for this surface: originally, investigations assigned a peak at ≈529.5 eV to oxygen in a twofold coordinated bridge site[2], while more recent investigations reassign this lower BE species to oxygen in hollow sites and a higher BE feature ≈530.6 eV as oxygen at bridge sites[3,4]. We use the more updated assignment for our experimental averages. Although there is a high error for DFT-calculated BEs for this species, it does not appear

that the older assignment would entirely resolve this discrepancy because no DFT study has identified a ≈530.6 species.

Table S1. A summary of XPS results for surface structures of oxygen-containing molecular species on transition metals.

Host Metal	Facet/Structure	Bonded Species and Site	Coverage (ML)	Avg BE
Pt	(111) (2x2)[5]	NO *Hollows*	0.25[5]	530.5[5,6]
Ir	(111) √3x√3[7]	CO *Atop*	0.33[7]	532.1[8–10]
Ag	(111)	H_2O		533.3[11,12]
Pd	(111) c(2x4)[13]	CO *Hollows*	0.5[13]	531.2[14,15]
Rh	(111) 2x2[16]	CO *Atop*	~0.5[16]	532.1[17,18]
	(111) c(2x4)	CO *Hollows*		530.8[10,18,19]
	(111) c(2x4)[20]	NO *Hollows*	0.5[20]	530.9[20,21]
	(110) 2x2[22]	CO *Atop*	>0.5[22]	531.8[22–24]
Cu	(110) (2x1) [25]	CO	<0.5[25]	532.5[26–28]
Ni	(111) 2x2 [29]	CO *Atop*	~0.5[29]	532.3[29,30]
	(111) 2x2 [29]	CO *Hollows*	~0.5[29]	530.8[29]
	(111) 2x2 [29]	CO *Bridge*	~0.5[29]	531.1[29,30]

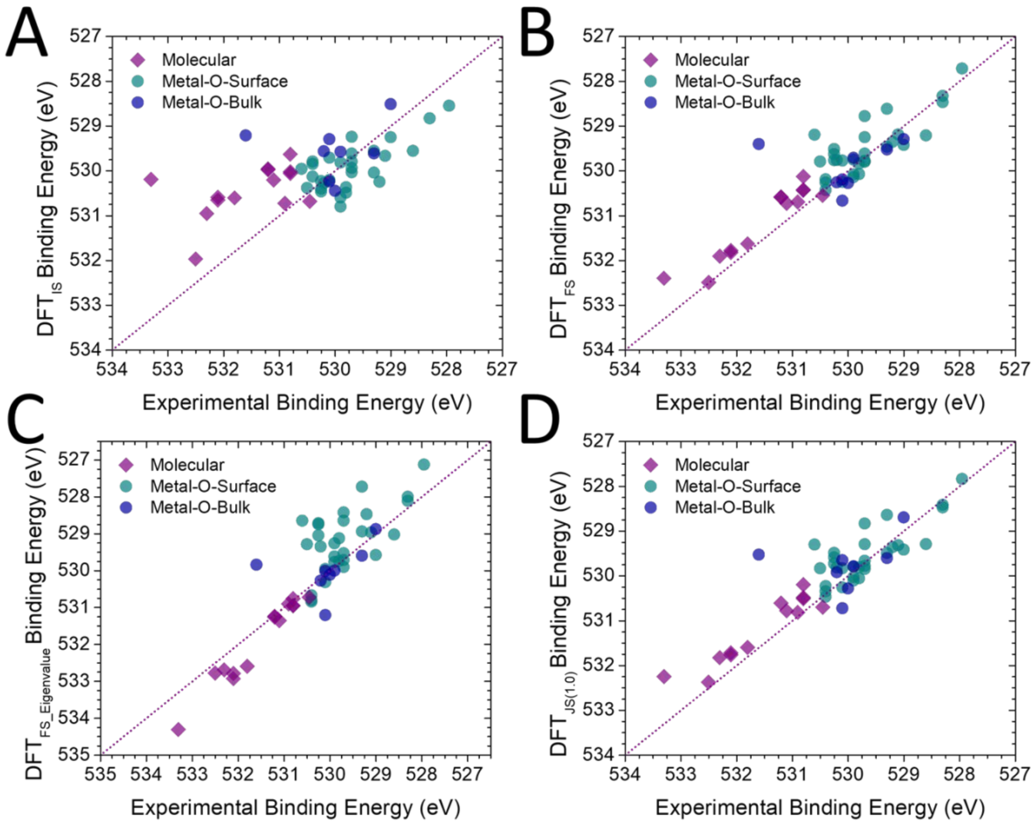

Figure S1. The accuracy of different DFT approximations in predicting the binding energies of molecular and atomic oxygen species. Experimentally determined BE values compared to DFT values using (A) initial state (B) final state (C) final state eigenvalues and (D) JS(1,0) approximations all reveal similar inconsistencies between predicted BEs for atomic oxygen species (circles) and more accurate predictions for molecular species (diamonds).

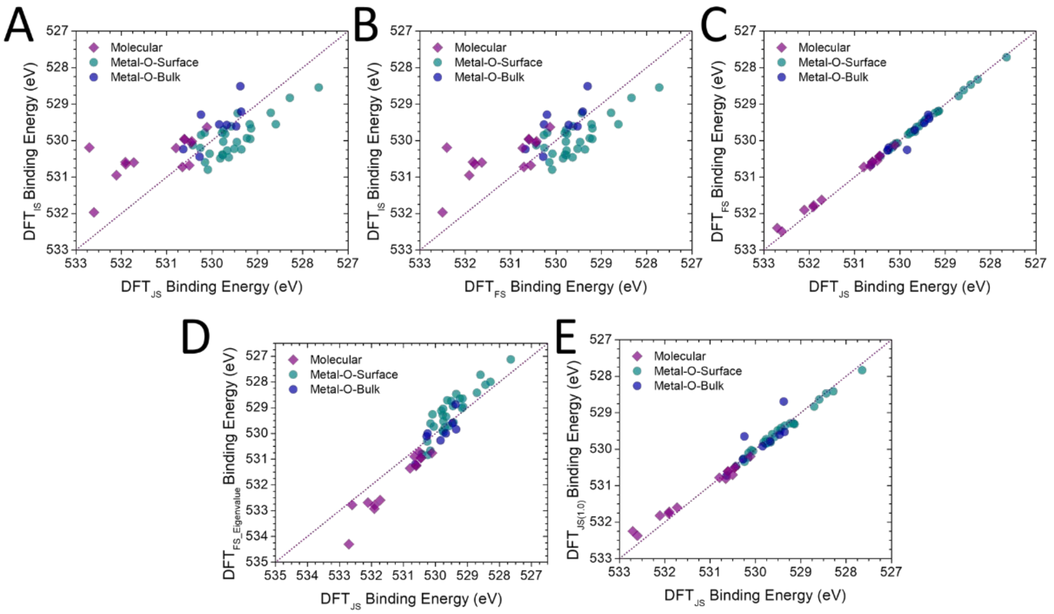

Figure S2. A comparison of binding energy predictions between Janak-Slater and other DFT approximations. Alternative approximations (A) initial state, (C) final state, (D) final state eigenvalues and (E) JS(1,0) compared to Janak-Slater tend to reveal minimal differences calculated BEs, with initial state approximations having the most varied results compared to both Janak-Slater and final state approximations (B).

References

(1) Jain, A.; Ong, S. P.; Hautier, G.; Chen, W.; Richards, W. D.; Dacek, S.; Cholia, S.; Gunter, D.; Skinner, D.; Ceder, G.; Persson, K. A. Commentary: The Materials Project: A Materials Genome Approach to Accelerating Materials Innovation. *APL Mater.* **2013**, *1* (1), 011002. https://doi.org/10.1063/1.4812323.

(2) Novotny, Z.; Tobler, B.; Artiglia, L.; Fischer, M.; Schreck, M.; Raabe, J.; Osterwalder, J. Kinetics of the Thermal Oxidation of Ir(100) toward IrO2 Studied by Ambient-Pressure X-Ray Photoelectron Spectroscopy. *J. Phys. Chem. Lett.* **2020**, *11* (9), 3601–3607. https://doi.org/10.1021/acs.jpclett.0c00914.

(3) Cao, X.; Liu, C.; Zhang, T.; Xu, Q.; Zhang, D.; Liu, X.; Jiao, H.; Wen, X.; Yang, Y.; Li, Y.; Niemantsverdriet, J. W.; Zhu, J. Revisiting Oxygen Adsorption on Ir(100). *J. Phys. Chem. C* **2022**, *126* (24), 10035–10044. https://doi.org/10.1021/acs.jpcc.2c01237.

(4) Martin, R.; Kim, M.; Lee, C. J.; Mehar, V.; Albertin, S.; Hejral, U.; Merte, L. R.; Lundgren, E.; Asthagiri, A.; Weaver, J. F. High-Resolution X-Ray Photoelectron Spectroscopy of an IrO2(110) Film on Ir(100). *J. Phys. Chem. Lett.* **2020**, *11* (17), 7184–7189. https://doi.org/10.1021/acs.jpclett.0c01805.

(5) Zhu, J. F.; Kinne, M.; Fuhrmann, T.; Denecke, R.; Steinrück, H.-P. In Situ High-Resolution XPS Studies on Adsorption of NO on Pt(111). *Surf. Sci.* **2003**, *529* (3), 384–396. https://doi.org/10.1016/S0039-6028(03)00298-X.

(6) Shimada, T.; Mun, B. S.; Nakai, I. F.; Banno, A.; Abe, H.; Iwasawa, Y.; Ohta, T.; Kondoh, H. Irreversible Change in the NO Adsorption State on Pt(111) under High Pressure Studied by AP-XPS, NEXAFS, and STM. *J. Phys. Chem. C* **2010**, *114* (40), 17030–17035. https://doi.org/10.1021/jp102777j.

(7) Lauterbach, J.; Boyle, R. W.; Schick, M.; Mitchell, W. J.; Meng, B.; Weinberg, W. H. The Adsorption of CO on Ir(111) Investigated with FT-IRAS. *Surf. Sci.* **1996**, *350* (1), 32–44. https://doi.org/10.1016/0039-6028(95)01114-5.

(8) Zhdan, P. A.; Boreskov, G. K.; Boronin, A. I.; Schepelin, A. P.; Withrow, S. P.; Weinberg, W. H. An XPS Investigation of CO Titration of Oxygen from an Ir(111) Surface. *Appl. Surf. Sci.* **1979**, *3* (2), 145–160. https://doi.org/10.1016/0378-5963(79)90015-1.

(9) Zhdan, P. A.; Boreskov, G. K.; Egelhoff, W. F.; Weinberg, W. H. The Application of XPS to the Determination of the Kinetics of the Co Oxidation Reaction over the Ir(111) Surface. *Surf. Sci.* **1976**, *61* (2), 377–390. https://doi.org/10.1016/0039-6028(76)90052-2.

(10) Ueda, K.; Isegawa, K.; Amemiya, K.; Mase, K.; Kondoh, H. Operando NAP-XPS Observation and Kinetics Analysis of NO Reduction over Rh(111) Surface: Characterization of Active Surface and Reactive Species. *ACS Catal.* **2018**, *8* (12), 11663–11670. https://doi.org/10.1021/acscatal.8b03180.

(11) Felter, T. E.; Weinberg, W. H.; Lastushkina, G. Ya.; Zhdan, P. A.; Boreskov, G. K.; Hrbek, J. The Adsorption of Methanol on Ag(111) and Its Reaction with Preadsorbed Oxygen. *Appl. Surf. Sci.* **1983**, *16* (3), 351–364. https://doi.org/10.1016/0378-5963(83)90079-X.

(12) Schwaner, A. L.; White, J. M. Electron-Induced Chemistry of Methanol on Ag(111). *J. Phys. Chem. B* **1997**, *101* (49), 10414–10422. https://doi.org/10.1021/jp972136y.

(13) Surnev, S.; Sock, M.; Ramsey, M. G.; Netzer, F. P.; Wiklund, M.; Borg, M.; Andersen, J. N. CO Adsorption on Pd(111): A High-Resolution Core Level Photoemission and Electron Energy Loss Spectroscopy Study. *Surf. Sci.* **2000**, *470* (1–2), 171–185. https://doi.org/10.1016/S0039-6028(00)00853-0.

(14) Toyoshima, R.; Yoshida, M.; Monya, Y.; Kousa, Y.; Suzuki, K.; Abe, H.; Mun, B. S.; Mase, K.; Amemiya, K.; Kondoh, H. In Situ Ambient Pressure XPS Study of CO Oxidation Reaction on Pd(111) Surfaces. *J. Phys. Chem. C* **2012**, *116* (35), 18691–18697. https://doi.org/10.1021/jp301636u.

(15) Martin, N. M.; Van den Bossche, M.; Grönbeck, H.; Hakanoglu, C.; Zhang, F.; Li, T.; Gustafson, J.; Weaver, J. F.; Lundgren, E. CO Adsorption on Clean and Oxidized Pd(111). *J. Phys. Chem. C* **2014**, *118* (2), 1118–1128. https://doi.org/10.1021/jp410895c.

(16) Schwegmann, S.; Over, H.; De Renzi, V.; Ertl, G. The Atomic Geometry of the O and CO + O Phases on Rh(111). *Surf. Sci.* **1997**, *375* (1), 91–106. https://doi.org/10.1016/S0039-6028(97)01249-1.
(17) Wagner, F. T.; Moylan, T. E.; Schmieg, S. J. Hydrophilic versus Hydrophobic Coadsorption: Carbon Monoxide and Water on Rh(111) versus Pt(111). *Surf. Sci.* **1988**, *195* (3), 403–428. https://doi.org/10.1016/0039-6028(88)90350-0.
(18) DeLOUISE, L. A.; White, E. J.; Winugrad, N. Characterization of CO Binding Sites on Rh(111) and Rh(331) Surfaces by XPS and LEED: Comparison to EELS Results.
(19) DeLouise, L. A.; Winograd, N. Carbon Monoxide Adsorption and Desorption on Rh{111} and Rh{331} Surfaces. *Surf. Sci.* **1984**, *138* (2), 417–431. https://doi.org/10.1016/0039-6028(84)90256-5.
(20) Toyoshima, R.; Yoshida, M.; Monya, Y.; Suzuki, K.; Amemiya, K.; Mase, K.; Mun, B. S.; Kondoh, H. High-Pressure NO-Induced Mixed Phase on Rh(111): Chemically Driven Replacement. *J. Phys. Chem. C* **2015**, *119* (6), 3033–3039. https://doi.org/10.1021/jp507542h.
(21) DeLouise, L. A.; Winograd, N. Adsorption and Dessorption of NO from Rh(111) and Rh(331) Surfaces. *Surf. Sci.* **1985**, *159*, 199–213.
(22) Dhanak, V. R.; Baraldi, A.; Comelli, G.; Paolucci, G.; Kiskinova, M.; Rosei, R. CO Adsorption on Unreconstructed and Reconstructed Rh(110) Surfaces: LEED and XPS Studies. *Surf. Sci.* **1993**, *295* (3), 287–294. https://doi.org/10.1016/0039-6028(93)90275-O.
(23) Nguyen, L.; Liu, L.; Assefa, S.; Wolverton, C.; Schneider, W. F.; Tao, F. F. Atomic-Scale Structural Evolution of Rh(110) during Catalysis. *ACS Catal.* **2017**, *7* (1), 664–674. https://doi.org/10.1021/acscatal.6b02006.
(24) Baird, R. J.; Ku, R. C.; Wynblatt, P. The Chemisorption of CO and NO on Rh(110). *Surf. Sci.* **1980**, *97* (2), 346–362. https://doi.org/10.1016/0039-6028(80)90672-X.
(25) Hofmann, Ph.; Schindler, K.-M.; Bao, S.; Fritzsche, V.; Bradshaw, A. M.; Woodruff, D. P. A Photoelectron Diffraction Study of the Structure of the Cu{110}(2 × 1)-CO System. *Surf. Sci.* **1995**, *337* (3), 169–176. https://doi.org/10.1016/0039-6028(95)00612-5.
(26) Eren, B.; Lichtenstein, L.; Wu, C. H.; Bluhm, H.; Somorjai, G. A.; Salmeron, M. Reaction of CO with Preadsorbed Oxygen on Low-Index Copper Surfaces: An Ambient Pressure X-Ray Photoelectron Spectroscopy and Scanning Tunneling Microscopy Study. *J. Phys. Chem. C* **2015**, *119* (26), 14669–14674. https://doi.org/10.1021/jp512831f.
(27) Christiansen, M.; Thomsen, E. V.; Onsgaard, J. Coadsorption of K and CO on Cu(110). *Surf. Sci.* **1992**, *261* (1), 179–190. https://doi.org/10.1016/0039-6028(92)90230-4.
(28) Gruzalski, G. R.; Zehner, D. M.; Wendelken, J. F. An XPS Study of Oxygen Adsorption on Cu(110). *Surf. Sci.* **1985**, *159* (2), 353–368. https://doi.org/10.1016/0039-6028(85)90433-9.
(29) Held, G.; Schuler, J.; Sklarek, W.; Steinrück, H.-P. Determination of Adsorption Sites of Pure and Coadsorbed CO on Ni(111) by High Resolution X-Ray Photoelectron Spectroscopy. *Surf. Sci.* **1998**, *398* (1), 154–171. https://doi.org/10.1016/S0039-6028(98)80020-4.
(30) Degerman, D.; Lömker, P.; Goodwin, C. M.; Shipilin, M.; García-Martínez, F.; Schlueter, C.; Nilsson, A.; Amann, P. State of the Surface During CO Hydrogenation over Ni(111) and Ni(211) Probed by Operando X-Ray Photoelectron Spectroscopy. *J. Phys. Chem. C* **2023**, *127* (8), 4021–4032. https://doi.org/10.1021/acs.jpcc.2c07650.
